# Organoleptic Analysis of Drinking Water Using an Electronic Tongue Based on Electrochemical Microsensors

**DOI:** 10.3390/s19061435

**Published:** 2019-03-23

**Authors:** Manuel Gutiérrez-Capitán, Marta Brull-Fontserè, Cecilia Jiménez-Jorquera

**Affiliations:** 1 Instituto de Microelectrónica de Barcelona (IMB-CNM), CSIC. Campus de la UAB, 08193 Bellaterra, Barcelona, Spain; cecilia.jimenez@csic.es; 2 Mina Pública d’Aigües de Terrassa, S.A. C/de la Societat, 26, 08221 Terrassa, Barcelona, Spain; mbrull@aiguesdeterrassa.com

**Keywords:** organoleptic tests, electrochemical microsensors, electronic tongue, multivariate methods, drinking water samples

## Abstract

The standards that establish water’s quality criteria for human consumption include organoleptic analysis. These analyses are performed by taste panels that are not available to all water supply companies with the required frequency. In this work, we propose the use of an electronic tongue to perform organoleptic tests in drinking water. The aim is to automate the whole process of these tests, making them more economical, simple, and accessible. The system is composed by an array of electrochemical microsensors and chemometric tools for multivariable processing to extract the useful chemical information. The array of sensors is composed of six Ion-Sensitive Field Effect Transistors (ISFET)-based sensors, one conductivity sensor, one redox potential sensor, and two amperometric electrodes, one gold microelectrode for chlorine detection, and one nanocomposite planar electrode for sensing electrochemical oxygen demand. A previous study addressed to classify water samples according to taste/smell descriptors (sweet, acidic, salty, bitter, medicinal, chlorinous, mouldy, and earthy) was performed. A second study comparing the results of two organoleptic tests (hedonic evaluation and ranking test) with the electronic tongue, using Partial Least Squares regression, was conducted. The results show that the proposed electronic tongue is capable of analyzing water samples according to their organoleptic characteristics, which can be used as an alternative method to the taste panel.

## 1. Introduction

Quality control of drinking water is associated with the analysis of different parameters, and organoleptic characteristics [1]. The organoleptic quality is defined as the result of evaluating water based on smell, taste, color, and turbidity. If the water has an unusual taste or smell (or it is cloudy or colored), it can be interpreted as a health risk and a problem in the water source, its treatment, or in the water network. Therefore, water companies are aware that people are becoming more demanding regarding the sensory quality of water.

The methodology for analyzing the sensory quality of water is based on the use of descriptors. This is the technical term used to define the characteristics that are perceived by the senses of taste and smell in the presence of certain compounds. Determining chemical substances related to the different descriptors in water can present analytical difficulties because they are often present at very low concentrations. They can also come from different sources. For example, geosmin and 2-methylisoborneol compounds related with earthy and mouldy descriptors, respectively, are both released by microorganisms such as actinomycetes or cyanobacteria. Other substances such as sodium chloride have a natural mineral origin related with the salty descriptor, or an anthropogenic origin, such as the pollutant dichlorophenol, which produces a medicinal flavor. Others are produced during the potabilization processes, such as chlorine, which is added to water as a disinfectant [2].

Sensory tests are performed by a group of people who are taste panel trained for identifying, differentiating, and quantifying the smells and tastes of water [3]. According to the objective pursued, there are four types of tests that can be performed by the taste panel. Descriptive tests, which objective is to identify and quantify the descriptors of water; threshold flavor test, used to determine the threshold of flavor by diluting the sample with flavor-free water until the minimum perceptible flavor is achieved; discriminating tests, that enable the detection of significant differences between samples or between them and a reference sample; and acceptance tests, where the taste panel ranks or classifies the samples in terms of their preference or their level of satisfaction upon tasting them [4,5].

The minimum number of analyses established by the European Union Directive depends on the volume of water distributed or produced per day within a supply zone [1]. However, each Member State adapts the frequency of the organoleptic tests to its own legislation. In the case of Spain, these tests should be performed at least twice a week [6]. For large water supplier companies equipped with laboratories for water control, these tests are a routine task. However, for suppliers of medium and small towns, the cost of performing them in external facilities with the frequency required is not affordable. In fact, according to the official annual report of 2016 [7], in Spain less than 36% of the organoleptic analyses required by legislation were reported. Therefore, an analytical system able to automate these sensory tests and make them cheaper, simpler, and more accessible is of great interest.

One alternative is the use on an Electronic Tongue: an array of sensors for liquids with partially selective responses coupled with multivariate chemometric tools [8,9]. The advantages of these systems in comparison with human panels include higher objectivity and invariable response with time, which contribute to the success of routine analysis. As a bio-inspired approach, these analytical systems allow pattern recognition of characteristic signals for a particular taste. To date, many different electronic tongues have been reported for determining organoleptic characteristics of foodstuffs such as extra virgin olive oil [10], pomegranate juice [11], probiotic fermented milk [12], wines [13,14], apple juice [15], extracts from raw and dried apples [16], table olive [17], rice wine [18], beer [19], coke drinks [20], black tea [21] and even of pharmaceutical formulations [22]. For the taste analysis of drinking water, the literature reported is limited to a few papers. Of note, a study by Corvinus University of Budapest used the commercial electronic tongue ASTREE II (Alpha MOS, Toulouse, France) to classify mineral, spring, and tap water samples from different geographical origins and brands using Principal Component Analysis (PCA) and Linear Discriminant Analysis (LDA) [23,24]. In addition, accurate Partial Least Squares (PLS) models for the prediction of 11 sensory attributes in flavored mineral water samples were obtained by correlating the ASTREE II results with the scores from a trained human panel. These sensory attributes were both odor- and taste-related, and they were determined with low errors of prediction by the ASTREE electronic tongue. In fact, the lowest error was obtained for global taste intensity, which represents the contribution of all the taste components [25].

In the current work, we propose the use of an electronic tongue as an alternative method to the taste panel. To the best of our knowledge, this is the first time that a prediction study of organoleptic tests for drinking water from different origins is performed using an electronic tongue. The array of electrochemical sensors, fabricated with microelectronic technology, is composed of six Ion-Sensitive Field Effect Transistors (ISFET) sensors selective to pH and several common ions, one conductivity sensor, one redox potential (ORP) sensor, and two amperometric electrodes, one gold microelectrode for chlorine detection and one nanocomposite planar electrode for sensing electrochemical oxygen demand (EOD) [26]. To extract the chemical information, the obtained data are analyzed with PCA for the classification of synthetic water samples spiked with different taste/smell component descriptors, and for the patterning the recognition of real drinking water samples. Furthermore, two organoleptic tests were conducted by a taste panel in order to evaluate the correlation with the electronic tongue results using PLS regression. The results demonstrate the capability of the proposed system to classify water samples according to their organoleptic characteristics.

## 2. Materials and Methods

### 2.1. Reagents and Solutions

All reagents used were of high purity, analytical grade or equivalent. All solutions were prepared with de-ionized water from the IMB-CNM facilities. For ISFET calibration, stock solutions with ionic salts of concentrations of 10^−4^, 10^−2^, and 1.0 M were prepared. In the case of those sensitive to Na^+^ and Ca^2+^ cations, the corresponding chloride salts were used. For Cl^−^, CO_3_^2−^ ISFETs and the generic to anions, solutions of NaCl, NaHCO_3_ and KNO_3_, respectively, were prepared. For the pH ISFET calibration, a universal buffer solution containing 0.04 M boric acid, 0.04 M acetic acid, 0.04 M phosphoric acid, and 0.1 M KNO_3_ was prepared. For the amperometric gold electrode, a solution containing 0.1 M KNO_3_ was used for electrochemical activation, and a 0.2 M PBS pH 5.5 solution was prepared for the water analysis. In order to calibrate the conductivity sensor, two different standard solutions from Crison (Hach Lange, Barcelona, Spain), with nominal values of 1,413 and 147 µS/cm (at 25 °C), were utilized. Two standard redox solutions from Panreac (Barcelona, Spain), with values of 468 and 220 mV (at 25 °C vs. Ag/AgCl) were used to test the ORP sensor. Finally, for the analysis with the electrochemical oxygen demand electrode, a 0.2 M NaOH solution was prepared.

### 2.2. Synthetic Water Samples

First we performed a study to evaluate the electronic tongue’s response in synthetic water samples. A solution without any taste or smell (flavor-free water) was used as background in all the synthetic samples. The chemical composition of this solution is shown in Table 1, based on a 1:1 mixture of water from the plant of reverse osmosis (RO) of the water supplier company “Mina Pública d’Aïgues de Terrassa, S.A.” (*Aïgues de Terrassa*), and water from the outlet of the drinking water treatment plant (DWTP) located in *Abrera* (Barcelona, Spain). To study the organoleptic characteristics, eight chemical substances were used as taste/smell descriptors—these substances are commonly used by taste panels as reference standards, although not all of them have been identified in real events of flavors detected in water [3]. Two levels of concentration were set for each descriptor and cross-effects were not considered. The high level related to the taste/smell threshold of concentration that establishes the Flavor Profile Analysis [4], while the low level is half of that threshold. Thus, 16 synthetic samples were prepared as shown in Table 2. These samples were prepared with the flavor-free water as a background, therefore they all have at least 200 mg/L NaCl.

### 2.3. Drinking Water Samples

The system was also evaluated with real samples of drinking water, provided by *Aigües de Terrassa*. The set consisted of 15 samples, which showed a great variability in order to ensure a broad experimental domain. In fact, these waters can be classified into four different groups:

- Two bottled natural waters, from *Aigua de Ribes* (Manantial Fontaga S.A., Ribes de Freser, Girona, Spain) and *Font Vella* (Aguas Danone S.A., Sant Hilari Sacalm, Girona, Spain).
- Mixtures of water from the RO plant and water from the outlet of DWTP, at four different proportions: 25% RO + 75% DWTP, 50% RO + 50% DWTP, 75% RO + 25% DWTP, and 100% DWTP.
- Tap waters from seven sampling points; two of them at the entrance to the distribution network (*Llobregat* river origin and *Mina* from the public water mine of Terrassa), and five different towns within the network (*Viladecavalls*, *Rellinars*, *Cardona*, *Vacarisses* and *Les Fonts*), all located in the province of Barcelona, Spain.
- Two waters prepared by mixture of the above: 50% *Mina* + 50% *Cardona* and 75% *Llobregat* + 25% *Aigua de Ribes*.

All these waters were previously analyzed by an accredited laboratory of the water supplier company (Laboratori Ambiental, Mina Pública Aigües de Terrassa, Terrassa, Spain) using standard methods [4]. The following chemical parameters were analyzed: nitrate, chloride and sulphate were determined by ionic chromatography; sodium, potassium, magnesium, and calcium were determined by inductively-coupled plasma optical emission spectrometry (ICP-OES); combined chlorine, free chlorine, and total chlorine were determined by UV-Vis spectrophotometry; bicarbonate and alkalinity were determined by acid-base titration; hardness was calculated from the calcium and magnesium concentrations; pH was determined by potentiometry using a glass-electrode, conductivity was determined by conductometry and Langelier index was calculated from the pH, temperature, hardness, and alkalinity values (Table 3). These parameters classify samples according to their chemical composition and allow for comparison of the results with those from the electronic tongue.

### 2.4. Organoleptic Analysis by a Taste Panel

These waters were also tested organoleptically at *Aigües de Terrassa* by a panel formed of 14 members, who have a great deal of experience as water tasters. Following an internal procedure, the samples were kept at 25 °C, and each panel member took 50 mL in a glass. The taster then took a small amount in his/her mouth, passing it over the tongue surface. After a few seconds, the water was expelled into a recipient and the results were noted down. For this study, two acceptance tests were performed by the panel: a ranking test, where the samples were ranked (from the first to the fifth), taking the taster’s preference as the only criterion; and a hedonic evaluation, where the tasters are asked to score their satisfaction upon tasting on a scale from 1 (Dislike extremely) to 10 (Like extremely). The tasting was carried out over three days, using five water samples with different chemical compositions each time. The first day, the samples tested were *Aigua de Ribes*, *Les Fonts*, *Vacarisses*, *Mina* and *Llobregat*; the second day *Rellinars*, *Cardona*, 50% RO + 50% DWTP, 25% RO + 75% DWTP, and 100% DWTP; the third day *Font Vella*, 75% RO + 25% DWTP, 50% *Mina* + 50% *Cardona*, 75% *Llobregat* + 25% *Ribes*, and *Viladecavalls*. With the 14 values for each sample and test, the mean was calculated.

### 2.5. Microsensors and Equipment

A set of six ISFET sensors were fabricated using standard microelectronic technology [27]. One ISFET was used for measuring pH and the rest were modified with polymeric membranes sensitive to Na^+^, Ca^2+^, Cl^−^, CO_3_^2−^, and generic for anions. Polymeric membranes were prepared with photocurable polymers (Ebecryl^®^, Allnex, Waalwijk, The Netherlands) and ionophores from Sigma-Aldrich (St. Louis, MO, USA). The ionophores used were: 4-tert-butylcalix [4] arenetetraacetic acid tetraethyl ester (Ionophore X) for Na^+^, N,N,N′,N′-tetracyclohexyl-3-oxapentanediamide (Ionophore II, ETH 129) for Ca^2+^, tridodecylmethylammonium chloride for Cl^−^, 4-butyl-α,α,α-trifluoroacetophenone (Ionophore IV) for CO_3_^2−^, and tetraoctylammonium bromide for generic response to anions. All these ionophores are not specific to its principal ion and they present a certain degree of cross-response to other ions in solution. Membrane composition, preparation, and characterization have been presented elsewhere [28,29,30,31]. An Orion 90-02-00 double junction Ag/AgCl reference electrode (Thermo Electron, Waltham, MA, USA) with 0.1 M lithium acetate solution in its outer chamber was employed for all the ISFET measurements. These were performed with a home-made readout circuit with the support of a data-acquisition system connected to a PC. For data acquisition and recording, a specially designed software programmed with VisualBasic (Microsoft Inc., Seattle, WA, USA) was used.

Two platinum-based microsensors were used as ORP and conductivity sensors. In the case of the conductivity, the microsensor had a four-electrode configuration. Their fabrication and characterization are reported elsewhere [32]. For signal conditioning and control data acquisition, a versatile and portable system developed at the IMB-CNM was used. This system was connected to the PC through a USB link. The control program was based on LabView graphic language (National Instruments, Austin, TX, USA).

Two different amperometric microsensors were employed: a conventional gold electrode fabricated according to standard photolithographic techniques and a nanocomposite planar electrode for sensing electrochemical oxygen demand (EOD) developed by our group [33]. In both cases, the amperometric cell contained the working electrode, a platinum commercial electrode as counter electrode (Radiometer, Lyon, France) and an Ag/AgCl/10% (w/v) KNO_3_ reference electrode (Metrohm 0726 100, Herisau, Switzerland). A µAutolab potentiostat/galvanostat (Ecochemie, Utrecht, The Netherlands), using NOVA 2.1 software package was used for all amperometric measurements.

### 2.6. Measurement Methodology

All the microsensors that form the array were calibrated before the analysis in order to check their correct functioning. Briefly, the ISFETs were calibrated by adding accumulated microvolumes of stock primary-ion solutions (10^−4^, 10^−2^, and 1.0 M) in a fixed volume of de-ionized water. In the case of the pH ISFET, microvolumes of 1 M NaOH solution were added to the universal buffer solution to change the pH from 2 to 12. In the case of the conductivity and ORP microsensors, they were calibrated using two standard solutions of 1413 and 147 µS/cm, and 220 and 468 mV, respectively. For the amperometric gold microelectrode, the calibration consisted of adding accumulated microvolumes of a stock solution of free chlorine (in the form of NaClO) in a 0.1 M PBS pH 5.5 solution. Finally, in the case of the EOD sensor, microvolumes of 25 g/L glucose stock solution were added to a 0.1 M NaOH solution to obtain the calibration curve. The response characteristics obtained are reported elsewhere [34].

The analysis of water samples was carried out under batch conditions at room temperature (around 22 °C). A complete analysis of a sample took around 10 min. No replicates for each sample were done to get a rapid analysis and prevent changes in the chemical composition of the water sample. The six ISFETs were immersed directly in the water sample and, after 3 min for signal stabilization, the potentials (in mV vs. Ag/AgCl) were recorded. The output values corresponding to the relative signal of the ISFET with respect to the flavor-free water solution (Table 1)—which was checked periodically—were used as analytical signals for the models. This is a common strategy to correct for the possible drift of the ISFET sensors. The conductivity and ORP microsensors were also immersed in the sample and after 3 min for stabilization, the signals (in mS/cm and mV, respectively) were recorded. In the case of the amperometric gold microelectrode, a 1:1 dilution of the water sample with 0.2 M PBS pH 5.5 was performed to adjust the ionic strength and pH for the optimal detection of free chlorine. By setting the potential at +350 mV (vs. Ag/AgCl), the chronoamperometric signal (in A) was recorded after 30 s on this dilution to detect the electrochemical reduction of free chlorine [35]. In the case of the nanocomposite microelectrode, a 1:1 dilution of the sample with 0.2 M NaOH was used to adjust the pH to 12.8 ± 0.5 for the optimal operation of the electrocatalytic EOD sensor. Using the chronoamperometric mode and setting the potential at +600 mV (vs. Ag/AgCl), the current signal (in A) was obtained after 30 s on this dilution to oxidize organic compounds [33].

### 2.7. Data Treatment

Two different multivariate methods were used in this work, Principal Component Analysis (PCA) and Partial Least Squares (PLS) regression. First, a qualitative analysis of the 16 synthetic water samples was carried out using PCA in order to evaluate the discrimination capability of the electronic tongue. A PCA was also performed in order to classify the 15 drinking water samples according to their chemical composition referred to the 16 parameters determined by the accredited laboratory and to compare the results with those obtained with the electronic tongue. Second, PLS regression was used to quantify the results of the two organoleptic tests performed by the taste panel. For that, the mean values of these tests for the 15 drinking water samples were used as reference data to train the electronic tongue. The PLS-1 variant (one PLS model for each determination) was used in order to obtain more accurate predictions.

Both for PCA and PLS-1, the original values were previously autoscaled (all the variables were centered to the mean value and set to a variance ($\sigma^{2}$) equal to 1) to avoid that variables have different influence on the models. The formula used to perform this pretreatment is $x*\;=\left(x-\bar{x}\right)/\sigma$, where x∗ is the autoscaled value, x is the raw value, $\bar{x}$ is the mean value and σ is the standard deviation of the variable. In addition, the classical non-linear iterative (NIPALS) algorithm, together with the leave-one-out cross-validation (LOOCV) method (by using each time one sample as the validation set and the remaining samples as the training set), was used in the PLS-1 regressions. To control all these parameters and to obtain the multivariate models, the Unscrambler v.10.5 informatics package (CAMO ASA, Oslo, Norway) was used.

## 3. Results and Discussion

### 3.1. Input Variables for the Models

In order to obtain the maximum information from the samples, once the water samples were measured using the sensors, a data matrix was constructed with different variables used as inputs for the multivariate methods. This means that the different models were composed by 10 variables corresponding to all sensors used, as summarized in Table 4. The input data of the six ISFETs corresponded to the relative signal in mV of each ISFET with respect to the flavor-free water solution. This solution was measured as control every three water samples. With this methodology, the ISFET drift, as well as the matrix effects, were compensated. For the conductivity and ORP sensors, the absolute signal after 3 min recording was included in the data matrix. This time was set to obtain a stable signal for the ORP sensor. For the two amperometric microelectrodes, the current at a fixed potential after 30 s recording was used as variable data.

### 3.2. Classification of Synthetic Water Samples

**Figure 1:**
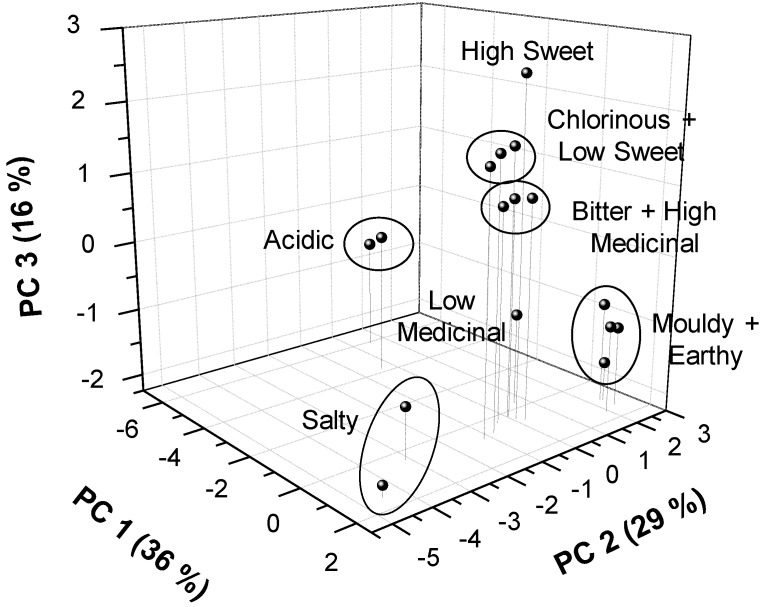
PCA score plot using the three first PC for the 16 synthetic water samples.

Water samples prepared with the different organoleptic descriptors (Table 2) were analyzed with the electronic tongue. The data matrix generated had a size of 10 × 16, which corresponded to 10 measured variables and 16 synthetic water samples. Figure 1 shows the results of the PCA model obtained. This 3D scores plot combined the information of the three first Principal Components (PC), which represent 81% of the total variance of the samples (36 + 29 + 16). In this model, the two acidic samples formed a separated group along PC 1, as well as the two salty samples along PC 2. This separation was due to contributions from the original variables showing that PC 1 is basically constituted by pH and CO_3_^2−^ ISFETs, whose primary ion is an acid–base species and the ORP sensor—which also contributes given the antioxidant properties of citric acid. In the case of PC 2, the variables with higher weight were the ISFET for Na^+^ and the conductivity sensor. In addition, the two mouldy samples and two earthy samples overlapped as there were no differences between them. These two substances were quite similar organoleptically and chemically (i.e., geosmin (C_12_H_22_O) is a bicyclodecane alcohol and 2-methylisoborneol (C_11_H_20_O) is a bicycloheptane alcohol), and they could interact in a similar way with the polymeric membrane of the ISFETs. The other synthetic samples were distributed along the PC 3, which correlated positively with the two amperometric variables, that is, the current at +350 mV, sensitive to free chlorine, and the current at +600 mV, sensitive to organic compounds. In fact, this is the reason why the two chlorinous samples and the solution with low sweet level overlapped. Another nearby group consists of the two bitter samples and the high medicinal level sample, which were nonetheless well separated from the low medicinal level sample. The sample with high sweet level was very well distinguished probably due to the high weight of the EOD variable in this PC 3. Therefore, this model can classify water according to the different descriptors used, especially those for which there are selective sensors in the electrochemical array (acidic, salty, chlorinous, and sweet).

### 3.3. Analysis of Drinking Water Samples

**Figure 2:**
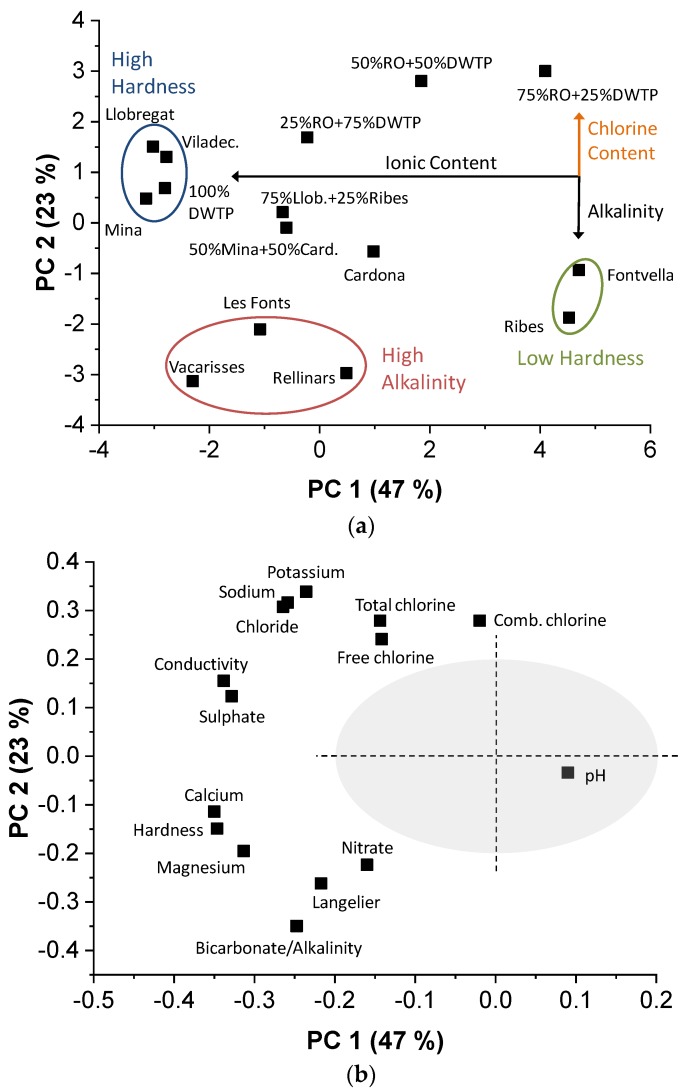
PCA (**a**) score plot and (**b**) loading plot obtained using the analytical data carried out by standard methods in the accredited laboratory. In the loading plot, the grey circle corresponds to the zone of low significance of the variables in the model. Llob.: *Llobregat*; Card.: *Cardona*; Viladec.: *Viladecavalls*; RO: outlet of reverse osmosis; DWTP: outlet of drinking water treatment plant.

The drinking water samples were measured with standard methods. The data matrix generated had a size of 16 × 15, which corresponded to 16 parameters determined in the accredited laboratory (values in Table 3) and 15 samples. Figure 2 represents the PCA model obtained with the data using standard analysis methods. The first two PC explain 70% of the variance for 15 real samples (47 + 23), according to their chemical composition. As can be seen in the score plot (Figure 2a), PC 1 differentiates the samples between those with High Hardness (*Llobregat*, *Mina*, *Viladecavalls* and 100%DWTP) and those with Low Hardness (two bottled natural waters). In addition, along this PC 1, the samples were distributed according to their ionic content. PC 2 separated according to alkalinity and chlorine content. Thus, tap water from *Les Fonts*, *Rellinars,* and *Vacarisses*, with high alkalinity values (246, 293, and 290 mg/L CaCO_3_, respectively), formed a group that differentiated from the rest. The sample 75% RO + 25% DWTP separated from the Low Hardness group due to its high free chlorine content (0.9 mg/L). Furthermore, the position of the two mixed samples (50% *Mina* + 50% *Cardona* and 75% *Llobregat* + 25% *Ribes*), halfway between the original waters that compose them. Finally, tap water from *Cardona* was the most neutral of this model, in terms of chemical composition, being the closest to the origin of the PCs.

This grouping and distribution of water samples were coherent with the loading plot (Figure 2b). However, the pH parameter was close to the origin (0, 0) and therefore, it is not significant to obtain a good classification model, given its narrow variation between samples (from 7.4 to 8.2, see Table 3). Analyzing the loadings in more detail, it was observed that PC 1 was constituted totally by ionic variables (conductivity, sulphate, calcium, etc.), while PC 2 mainly correlated positively with chlorine-related variables (free, combined, and total chlorine) and negatively with bicarbonate and alkalinity, which appeared superimposed with practically the same contribution in both PC 1 and PC 2.

**Figure 3:**
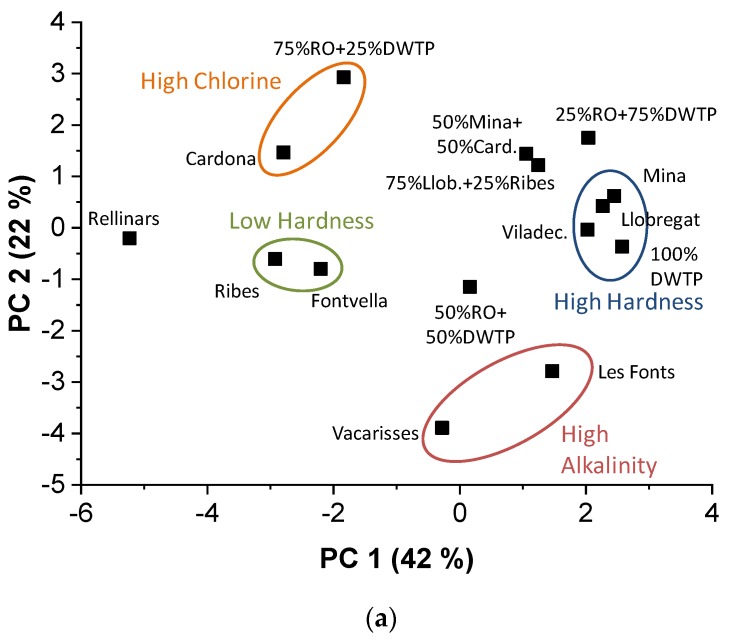

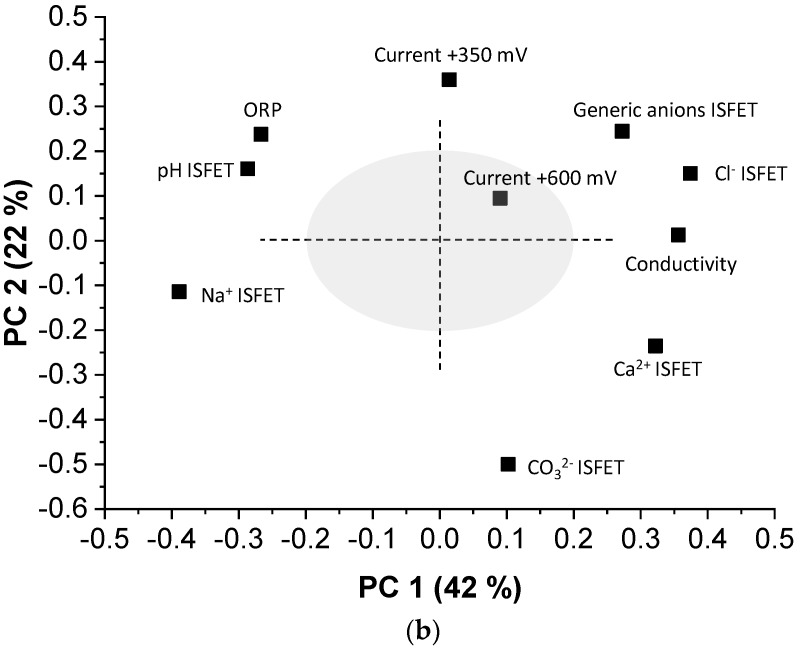
PCA (**a**) score plot and (**b**) loading plot obtained using the values of the variables from the electronic tongue. In the loading plot, the grey circle corresponds to the zone of low significance of the variables in the model. Llob.: *Llobregat*; Card.: *Cardona*; Viladec.: *Viladecavalls*; RO: outlet of reverse osmosis; DWTP: outlet of drinking water treatment plant.

These drinking water samples were also measured using the electronic tongue. The data matrix generated had a size of 10 × 15, which corresponded to 10 measured variables and 15 samples. Figure 3 represents the PCA model obtained from the electronic tongue. In this case, the first two PCs explained 64% of the total variance (42 + 22). By highlighting the groups of samples in the score plot (Figure 3a) with the same colors as in Figure 2a, we can compare the similarities using analytical data from standard methods and from the electronic tongue: orange for High Chlorine, red for High Alkalinity, blue for High Hardness, and green for Low Hardness. The last two groups related to hardness present a total coincidence between the two methods. The electronic tongue distinguished well the group of high total chlorine content that contains water from *Cardona* and 75% RO + 25% DWTP (0.9 and 1.0 mg/L, respectively). However, PCA for the standard methods did not include the *Cardona* sample. Regarding the high alkalinity group, the standard methods model grouped the samples from *Vacarisses*, *Les Fonts*, and *Rellinars*, but this last sample was not included in the electronic tongue model. In fact, the *Rellinars* sample had the lowest content of some ions, such as sodium (6 mg/L), potassium (0 mg/L), and nitrate (1.2 mg/L), for which there were variables in the electronic tongue model (ISFET sensors). According to the model with the electronic tongue, the sample 50% RO + 50% DWTP was the most neutral.

Analyzing the loading plot (Figure 3b) indicated that the current at +600 mV using the EOD sensor was not significant for the model, since no organic compounds were present for oxidation in these samples. As before, PC 1 was formed by ionic variables (conductivity, ISFETs for Cl^−^, Na^+^, Ca^2+^, pH, and generic to anions), but in this case they correlated mainly positively, which is the reason why the direction in which the ionic content increases is opposite in the two models. In the case of the PC 2, it mainly correlated positively with the current at +350 mV, related with the free chlorine, and negatively with the ISFET for CO_3_^2−^, related with the alkalinity; that is, the same correlation as the model with analytical data.

### 3.4. Correlation with the Taste Panel

**Figure 4:**
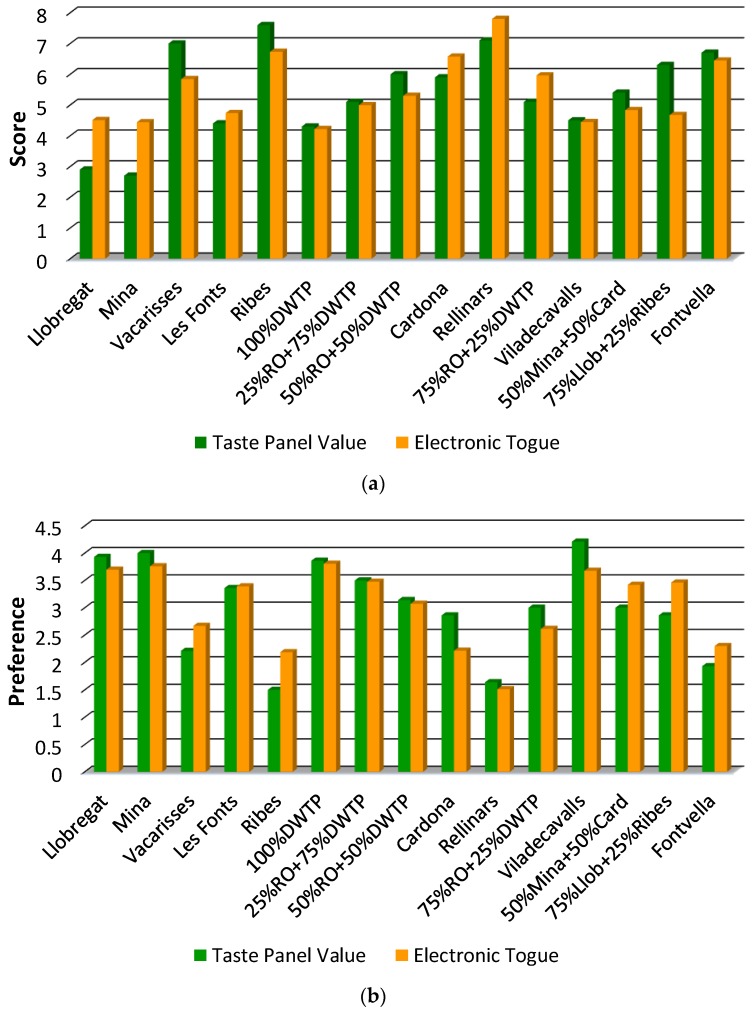
Bar graphs of (**a**) the hedonic evaluation (scoring the satisfaction upon tasting the samples on a scale from 1 (Dislike extremely) to 10 (Like extremely)), and (**b**) the ranking test (the samples are ranked from the first to the fifth, taking the preference as the only criterion) performed by the taste panel in comparison with the results obtained with the electronic tongue. Llob: *Llobregat*; Card: *Cardona*; RO: outlet of reverse osmosis; and DWTP: outlet of drinking water treatment plant.

Using the same experimental data as before (10 × 15 matrix dimensions), in Figure 4 are shown the results obtained with the taste panel and electronic tongue. As expected, the samples with a low score of satisfaction in the hedonic evaluation (Figure 4a) were less preferred in the ranking test (Figure 4b), and vice versa. This trend was also observed for both the taste panel and the electronic tongue. The values between these two methods were more similar in the case of the ranking test, probably due to the smaller range of variation of the response. In the case of the hedonic evaluation, there were discrepancies in the waters from *Llobregat* and *Mina*, since the tasting revealed values close to three, while the results obtained with the electronic tongue were above four. However, the 100% DWTP and *Viladecavalls* water samples, with a chemical composition very similar to *Llobregat* and *Mina* (see Table 3), the values were between 4 and 4.5 for both methods. On the other hand, tap water from *Rellinars*, according to the chemometric models, obtained better results of acceptance than those of the bottled waters.

A more detailed analysis of the numerical results showed a root-mean-square error (RMSE) of 0.9 units for the hedonic evaluation and 0.4 for the ranking test. This corresponded to a normalized RMSE close to 10%, considering the range of variation of the two analyses (scale from 1 to 10 for the hedonic evaluation, and from 1 to 5 for the ranking test). This value was within the range of error obtained in Reference [25] (0.80 < RMSE < 13.71, normalized to a scale from 0 to 100), in which the results of organoleptic analysis also correlated with those of an electronic tongue in flavored mineral water samples. However, it should be highlighted that tap drinking waters coming from the water network, without additives, were analyzed in the present work. In contrast, mineral water with artificially-added flavor or odor, exhibited more potentiated sensory attributes. Therefore it was easier, a priori, to determine the taste with a system like the electronic tongue, which responded to the changes of every tasty substance in water.

Finally, to demonstrate the applicability of the electronic tongue, a Student’s *t*-test for paired samples was applied to each of the acceptance tests. This statistical study allowed us to determine if there were systematic differences between the two different methods of measurement when the study was made with a set of real samples. This test did not consider only individual samples, and a *t*-statistic value of the whole set of differences was calculated. The experimental *t* values were 0.13 for the hedonic evaluation and 0.15 for the ranking test, while the tabulated value at a confidence level of 95% (*t*_tab,95%_^n-1=14^) was 2.14, confirming that the two methods were comparable.

## 4. Conclusions

The PCA model obtained for synthetic water samples demonstrated the viability of the electronic tongue in classifying the waters according to the different descriptors used, especially those for which there are selective sensors in the electrochemical array (Acidic, Salty, Chlorinous, and Sweet). Moreover, the obtained separation is also relevant for descriptors for which there are no sensors (Mouldy + Earthy, Bitter), thanks to the generalization capacity of the electronic tongue.

The PCA models using data from real drinking water samples obtained by standard methods and using the variables of the electronic tongue, showed that a very similar distribution and grouping of the waters, obtaining a good classification according to their chemical composition: hardness, alkalinity, chlorine content, and ionic content.

The interpolation of these real samples into PLS-1 models was to predict results from the taste panel, without statistically systematic errors. Specifically, a RMSE of 0.9 units for the hedonic evaluation and, of 0.4 for the ranking test were obtained. It is worthy to mention that, with this system, qualitative (PCA) and quantitative (PLS) analyses are performed using the same data, without additional experimental work. Furthermore, the results were not affected by external factors, whether physiological or psychological, that can influence the tasters and can invalidate the organoleptic tests.

Therefore, it can be concluded that the results show that the proposed electronic tongue is capable of analyzing water samples in order to classify them according to the organoleptic characteristics and thus become an alternative method to the taste panel for water supply companies and natural water bottling plants. However, the reliability of the system should be improved by processing a higher number of water samples.

## Figures and Tables

**Table 1:** Composition of the flavor-free water solution used as background.

Component	Concentration (mg/L)
NaCl	70
KNO_3_	20
MgSO_4_ × 7H_2_O	130
NaHCO_3_	170
CaCl_2_ × 2H_2_O	180

**Table 2:** Concentration levels of the chemical descriptors for the 16 synthetic water samples.

Descriptor	Substance	Concentration	
		Low Level	High Level
Sweet	Glucose	125 mg/L	250 mg/L
Acidic	Citric acid	125 mg/L	250 mg/L
Salty	Sodium chloride	500 mg/L	700 mg/L
Bitter	Caffeine	125 mg/L	250 mg/L
Medicinal	2,6-Dichlorophenol	100 ng/L	200 ng/L
Chlorinous	Free chlorine	0.25 mg/L	0.5 mg/L
Mouldy	2-Methylisoborneol	100 ng/L	200 ng/L
Earthy	Geosmin	150 ng/L	300 mg/L

**Table 3:** Values and concentrations of chemical parameters determined by an accredited laboratory (Laboratori Ambiental, Mina Puública Aiguües de Terrassa, Terrassa, Spain) using standard methods for the 15 drinking water samples.

**Drinking Water Samples**	**Nitrate** **(in mg/L)**	**Langelier** **Index**	**Combined Chlorine** **(in mg/L)**	**Free Chlorine** **(in mg/L)**	**Total Chlorine** **(in mg/L)**	**Chloride** **(in mg/L)**	**Conductivity** **(in µS/cm at 20 °C)**	**pH**
***Llobregat***	7.0	0.1	0.1	0.7	0.8	267	1363	7.6
***Mina***	13.0	0.2	0.1	0.6	0.7	249	1349	7.7
***Vacarisses***	16.0	0.0	0.1	0.5	0.6	37	836	7.4
***Les Fonts***	16.0	0.1	0.1	0.4	0.5	113	893	7.5
***Ribes***	11.0	−0.2	0.0	0.0	0.0	6	264	7.9
**100% DWTP**	6.7	0.3	0.1	0.4	0.5	275	1331	7.8
**25%RO + 75%DWTP**	5.6	−0.1	0.2	0.5	0.7	210	1023	7.8
**50%*Mina* + 50%*Cardona***	8.5	0.2	0.1	0.6	0.7	135	885	7.9
***Cardona***	4.0	0.5	0.1	0.8	0.9	43	550	8.2
***Rellinars***	1.2	0.5	0.1	0.5	0.6	11	549	7.9
**75%RO + 25%DWTP**	2.7	−1.1	0.1	0.9	1.0	78	396	7.6
***Viladecavalls***	8.1	0.1	0.1	0.7	0.8	258	1265	7.8
**50%RO + 50%DWTP**	3.7	−0.5	0.3	0.5	0.8	139	710	7.7
**75%*Llobregat* + 25%*Ribes***	7.7	0.2	0.1	0.4	0.5	188	1009	7.9
***Fontvella***	4.1	−0.3	0.1	0.0	0.0	16	275	7.9
**Uncertainty (%)**	19	-	-	19	14	17	4	4
**Drinking Water Samples**	**Sodium** **(in mg/L)**	**Sulphate** **(in mg/L)**	**Potassium** **(in mg/L)**	**Magnesium** **(in mg/L)**	**Calcium** **(in mg/L)**	**Bicarbonate** **(in mg/L)**	**Alkalinity** **(in mg/L CaCO_3_)**	**Hardness** **(in mg/L CaCO_3_)**
***Llobregat***	120	128	28	27	97	233	191	350
***Mina***	109	125	24	28	103	245	201	370
***Vacarisses***	18	123	2	41	100	354	290	414
***Les Fonts***	54	71	3	27	89	300	246	331
***Ribes***	7	20	1	6	40	138	113	124
**100% DWTP**	123	133	26	26	102	240	196	359
**25%RO + 75%DWTP**	87	98	15	19	73	171	140	259
**50%*Mina* + 50%*Cardona***	65	103	12	20	87	220	180	298
***Cardona***	22	83	2	14	78	214	176	251
***Rellinars***	6	19	0	28	77	357	293	305
**75%RO + 25%DWTP**	40	32	9	6	24	63	52	86
***Viladecavalls***	116	128	27	26	93	245	201	337
**50%RO + 50%DWTP**	68	66	15	13	50	118	97	177
**75%*Llobregat* + 25%*Ribes***	85	98	19	20	79	218	179	278
***Fontvella***	13	16	1	8	37	132	108	126
**Uncertainty (%)**	13	20	16	15	14	-	-	-

**Table 4:** Microsensors and variables used for constructing the multivariate models.

Microsensor	Variable
ISFET sensors	pH, Na^+^, Ca^2+^, Cl^−^, CO_3_^2−^ and generic for anions (in mV)
Pt electrode	ORP (in mV)
Pt 4-electrode	Conductivity (in mS/cm)
Gold electrode	Current at +350 mV (in A)
EOD sensor	Current at +600 mV (in A)

## References

[1] European Union (1998). Council Directive 98/83/EC of 3 November 1998 on the quality of water intended for human consumption. Official Journal of the European Communities.

[2] Fabrellas C., Cardenoso R., Devesa R., Flores J., Matia L. (2004). Taste and odor profiles (off-flavors) in the drinking waters of the Barcelona area (1996–2000). Water Sci. Technol..

[3] Devesa R., Fabrellas C., Cardenoso R., Matia L., Ventura F., Salvatella N. (2004). The panel of Aigues de Barcelona: 15 years of history. Water Sci. Technol..

[4] American Public Health Association (2005). APHA, AWWA, WEA. Standard Methods for the Examination of Water and Wastewater.

[5] European Commitee for Standardization, Water Quality (2006). Determination of the Threshold Odour Number (TON) and Threshold Flavour Number (TFN).

[6] Legislacion Consolidada (2003). RD 140/2003, de 7 de febrero, por el que se establecen los criterios sanitarios de la calidad del agua de consumo humano. Bolentín Oficial del Estado 45.

[7] Palau-Miguel M. (2016). Calidad del Agua de Consumo Humano en España. Informe Técnico.

[8] Holmberg M., Eriksson M., Krantz-Rülcker C., Artursson T., Winquist F., Lloyd-Spetz A., Lundström I. (2004). 2nd workshop of the second network on artificial olfactory sensing (NOSE II). Sens. Actuator B Chem..

[9] Vlasov Y., Legin A., Rudnitskaya A., di Natale C., D’Amico A. (2005). Nonspecific sensor arrays (“electronic tongue”) for chemical analysis of liquids (IUPAC Technical Report). Pure Appl. Chem..

[10] Dias L.G., Fernandes A., Veloso A.C.A., Machado A., Pereira J.A., Peres A.M. (2014). Single-cultivar extra virgin olive oil classification using a potentiometric electronic tongue. Food Chem..

[11] Bett-Garber K.L., Watson M.A., Lea J.M., Bai J.H., Baldwin E., Raithore S. (2014). Efficacy of monitoring the sensory taste characteristics in pomegranate juice with electronic tongue and chemical measurements. J. Food Qual..

[12] Hruskar M., Major N., Krpan M. (2010). Application of a potentiometric sensor array as a technique in sensory analysis. Talanta.

[13] Kirsanov D., Mednova O., Vietoris V., Kilmartin P.A., Legin A. (2012). Towards reliable estimation of an “electronic tongue” predictive ability from PLS regression models in wine analysis. Talanta.

[14] Rodríguez-Méndez M.L., de Saja J.A., Medina-Plaza C., García-Hernández C., Rodríguez-Méndez M.L. (2016). Chapter 26—Electronic tongues for the organoleptic characterization of wines. Electronic Noses and Tongues in Food Science.

[15] Kovacs Z., Sipos L., Szollosi D., Kokai Z., Szekely G., Fekete A. (2011). Electronic tongue and sensory evaluation for sensing apple juice taste attributes. Sens. Lett..

[16] Kutyla-Olesiuk A., Nowacka M., Wesoly M., Ciosek P. (2013). Evaluation of organoleptic and texture properties of dried apples by hybrid electronic tongue. Sens. Actuator B Chem..

[17] Marx I.M.G., Rodrigues N., Dias L.G., Veloso A.C.A., Pereira J.A., Drunkler D.A., Peres A.M. (2017). Assessment of table olives’ organoleptic defect intensities based on the potentiometric fingerprint recorded by an electronic tongue. Food Bioprocess Technol..

[18] Ouyang Q., Zhao J.W., Chen Q.S. (2014). Instrumental intelligent test of food sensory quality as mimic of human panel test combining multiple cross-perception sensors and data fusion. Anal. Chim. Acta.

[19] Rudnitskaya A., Polshin E., Kirsanov D., Lammertyn J., Nicolai B., Saison D., Delvaux F.R., Delvaux F., Legin A. (2009). Instrumental measurement of beer taste attributes using an electronic tongue. Anal. Chim. Acta.

[20] Szollosi D., Kovacs Z., Gere A., Sipos L., Kokai Z., Fekete A. (2012). Sweetener recognition and taste prediction of coke drinks by electronic tongue. IEEE Sens. J..

[21] Yaroshenko I., Kirsanov D., Kartsova L., Bhattacharyya N., Sarkar S., Legin A. (2014). On the application of simple matrix methods for electronic tongue data processing: Case study with black tea samples. Sens. Actuator B Chem..

[22] Woertz K., Tissen C., Kleinebudde P., Breitkreutz J. (2011). Taste sensing systems (electronic tongues) for pharmaceutical applications. Int. J. Pharm..

[23] Kovacs Z., Sipos L., Kantor D.B., Kokai Z., Fekete A., Pardo M., Sberveglieri G. (2009). Mineral water taste attributes evaluated by sensory panel and electronic tongue. Proceedings of the 13th International Symposium on Olfaction and Electronic Nose.

[24] Sipos L., Kovacs Z., Sagi-Kiss V., Csiki T., Kokai Z., Fekete A., Heberger K. (2012). Discrimination of mineral waters by electronic tongue, sensory evaluation and chemical analysis. Food Chem..

[25] Sipos L., Gere A., Szollosi D., Kovacs Z., Kokai Z., Fekete A. (2013). Sensory evaluation and electronic tongue for sensing flavored mineral water taste attributes. J. Food Sci..

[26] Giménez-Gómez P., Escudé-Pujol R., Capdevila F., Puig-Pujol A., Jiménez-Jorquera C., Gutiérrez-Capitán M. (2016). Portable electronic tongue based on microsensors for the analysis of cava wines. Sensors.

[27] Jiménez C., Bratov A., Abramova N., Baldi A., Grimes C.A., Dickey E.C., Pishko M.V. (2005). ISFET based sensors: Fundamentals and applications. Encyclopedia of Sensors.

[28] Artigas J., Beltran A., Jiménez C., Baldi A., Mas R., Domínguez C., Alonso J. (2001). Application of ion sensitive field effect transistor based sensors to soil analysis. Comput. Electron. Agric..

[29] Bratov A., Abramova N., Domínguez C. (2004). Investigation of chloride sensitive ISFETs with different membrane compositions suitable for medical applications. Anal. Chim. Acta.

[30] Makarychev-Mikhailov S., Goryacheva O., Mortensen J., Legin A., Levitchev S., Vlasov Y. (2003). Carbonate sensors based on 4-hexyltrifluoroacetophenone modified by acceptor substituents in phenyl ring. Electroanalysis.

[31] Isildak I., Asan A. (1999). Simultaneous detection of monovalent anions and cations using all solid-state contact PVC membrane anion and cation-selective electrodes as detectors in single column ion chromatography. Talanta.

[32] Orozco J., Baldi A., Baena R., Cadarso A., Bratov A., Jiménez C. (2007). Portable system based on microsensors for environmental monitoring applications. Meas. Sci. Technol..

[33] Gutiérrez-Capitán M., Baldi A., Gómez R., García V., Jiménez-Jorquera C., Fernández-Sánchez C. (2015). Electrochemical nanocomposite-derived sensor for the analysis of chemical oxygen demand in urban wastewaters. Anal. Chem..

[34] Gutiérrez M., Llobera A., Vila-Planas J., Capdevila F., Demming S., Buttgenbach S., Mínguez S., Jiménez-Jorquera C. (2010). Hybrid electronic tongue based on optical and electrochemical microsensors for quality control of wine. Analyst.

[35] Olive-Monllau R., Orozco J., Fernández-Sánchez C., Baeza M., Bartrolí J., Jiménez-Jorquera C., Céspedes F. (2009). Flow injection analysis system based on amperometric thin-film transducers for free chlorine detection in swimming pool waters. Talanta.

